# Clinical laboratory verification of thyroglobulin concentrations in the presence of autoantibodies to thyroglobulin: comparison of EIA, radioimmunoassay and LC MS/MS measurements in an Urban Hospital

**DOI:** 10.1186/s13104-017-3050-6

**Published:** 2017-12-08

**Authors:** Sarah E. Wheeler, Li Liu, Harry C. Blair, Richard Sivak, Nancy Longo, Jeffery Tischler, Kathryn Mulvey, Octavia M. Peck Palmer

**Affiliations:** 1 Department of Pathology, University of Pittsburgh School of Medicine, S723 Scaife Hall, 3550 Terrace St, Pittsburgh, PA 15261 USA; 2 University of Pittsburgh Medical Center (UPMC), Clinical Laboratory Building, 3477 Euler Way, Room 3014, Pittsburgh, PA 15213 USA; 3 Department of Critical Care Medicine, University of Pittsburgh School of Medicine, Pittsburgh, PA 15213 USA; 4 Department of Clinical and Translational Sciences, University of Pittsburgh School of Medicine, Pittsburgh, PA 15213 USA

**Keywords:** Thyroglobulin, Thyroglobulin antibody, Thyroid cancer, RIA, LC MS/MS

## Abstract

**Objective:**

Thyroglobulin (Tg) measurements assess recurrence in post-thyroidectomy thyroid cancer patients. Tg measurements by enzyme immunoassays (EIA) can be falsely elevated by interference from Tg autoantibodies (TgAb). Radioimmunoassay (RIA) is less susceptible to TgAb interference and has been the standard-of-care test for TgAb positive patients. Recently developed liquid chromatography tandem mass spectrometry (LC–MS/MS) methods may eliminate TgAb interference. We assessed the performance of Tg measurements by EIA, RIA and LC–MS/MS to evaluate TgAb interference differences.

**Results:**

We measured TgAb and Tg in 50 plasma samples from 40 patients in whom Tg measurement was part of their routine follow-up and 10 healthy volunteers. Discrepancy between EIA and both LC–MS/MS and RIA was observed at low Tg concentrations (≤ 7.55 ng/mL) in TgAb positive specimens (LC–MS/MS = 1.9 * EIA − 0.03, r = 0.68). RIA and LC–MS/MS Tg measurements in TgAb positive specimens with low Tg concentrations had improved correlation but demonstrated bias (LC MS/MS = 0.6 * RIA − 1.4, r = 0.90). Disagreement between methods may be attributed to LC–MS/MS reported Tg concentrations as undetectable compared to RIA. It seems likely that most discrepant cases are falsely elevated in RIA due to TgAb interference, however, some cases appear below the detection limit of LC–MS/MS; implementation of LC–MS/MS by clinicians will require lower detection limits.

**Electronic supplementary material:**

The online version of this article (10.1186/s13104-017-3050-6) contains supplementary material, which is available to authorized users.

## Introduction

The American Cancer Society estimates 56,870 individuals will be diagnosed with thyroid cancer in 2017 alone [1]. Differentiated thyroid cancer (DTC) accounts for most thyroid cancers and despite high survival rates with DTC, recurrence is very common (20–30% of patients will recur in their lifetime) [2, 3]. Standard of care following diagnosis with DTC is total thyroidectomy followed by radio iodine ablation to remove remnants and thyroid hormone replacement therapy [4]. Cancer recurrence is assessed annually by ultrasound imaging and thyroglobulin measurements [5–7].

Tg autoantibodies (TgAb) occur in approximately 25% of DTC patients [8], and common enzyme immunometric Tg assays (EIA) are susceptible to TgAb interference [9–13]. Tg radioimmunoassay methods are less susceptible to TgAb interference due to the competitive nature of the RIA and the use of polyclonal antibodies [10]. Recently, LC MS/MS Tg methods that may not be susceptible to TgAb interference are available [14–16].

Tg testing practices in our clinical laboratory consist of determination of the TgAb status. Specimens that are TgAb positive are sent to a reference laboratory for Tg analysis via RIA methodology while negative specimens are tested in-house for Tg via EIA. Analytical sensitivity is clinically necessary for early recurrence detection in patients followed for DTC. Ongoing evaluation of available Tg methods is important to provide improved standard of care to patients and we performed a multi-instrument method comparison study of EIA, RIA and LC MS/MS to consider altering our current testing algorithm. Using patient and healthy volunteer samples, we examined Tg measurements in both TgAb negative and positive specimens among the three methods. Specifically, TgAb positive specimens with low Tg concentrations (< 7.55 ng/mL) exhibited reduced correlation and increased bias among the EIA, RIA and LC MS/MS compared to correlation of all Tg concentrations.

## Main text

### Materials and methods

#### Specimens and study design

We used residual plasma from 40 patient specimens who had Tg testing as part of their routine clinical care at University of Pittsburgh Medical Center (UPMC) hospitals from June 2012 through May 2013. Samples were stored at − 80 °C until analysis. Specimens had the following in-house EIA Tg concentrations: < 0.1 ng/mL, N = 15; 0.1 and 2.0 ng/mL, N = 5; and Tg > 2.0 ng/mL, N = 20 (Additional file 1: Table S1, Additional file 2: Table S2).

Ten healthy adult volunteers were recruited from our clinical laboratory, provided written consent to donate a plasma specimen (age > 18 years; 9 females and 1 male), and received monetary compensation for time and specimen donation. Exclusion criteria for the healthy volunteers included pregnancy, diabetes, hospitalization in the past 30 days, current or history of thyroid disease (Graves or Hashimoto’s) or thyroid cancer, any cancers, known circulating autoantibodies, rheumatoid arthritis, or chronic medical complications.

Our study cohort (N = 50 specimens) was predominantly female (66%), Caucasian (72%) and 50% of the patients had papillary thyroid cancer (Additional file 1: Table S1). This study was approved by the UPMC Quality Improvement Review Committee.

#### Thyroglobulin antibody analysis

All 40 specimens were initially analyzed for TgAb concentrations using a solid phase enzyme labeled chemiluminescent sequential immunometric assay (Siemens Immulite 2000 XPi, Erlangen Germany; L2TGG12, L2TGA2) in-house (UPMC Presbyterian Hospital, Clinical Immunopathology, Pittsburgh, PA). The manufacturer has reported that TgAb > 20 IU/mL result in Tg assay interference. Patient specimens were divided into 2 groups and categorized as either: TgAb positive (N = 20; ≥ 20 IU/mL) or TgAb negative (N = 20; < 20 IU/mL).

Following TgAb status determination specimens were shipped to two separate reference laboratories for Tg measurements.

#### Thyroglobulin analysis

Tg concentrations were measured on all 50 specimens by in-house EIA, and by EIA and LC MS/MS at a reference laboratory (Additional file 2: Table S2). The LC MS/MS method was developed to detect Tg and overcome TgAb interference by trypsinization of the sera followed by enrichment of Tg peptides by immunoprecipitation before LC MS/MS [15].

Tg analysis by RIA was performed in only TgAb positive specimens (N = 20) at a reference laboratory [8, 17–19] as a part of our standard testing algorithm for TgAb positive patient specimens (Additional file 2: Table S2).

#### Statistical methods

Deming regression analysis was performed for method comparison studies per the standards of laboratory medicine. Low Tg is defined as less than or equal to the median Tg concentration of the 50 patient specimen cohort measured in-house. Statistical methods were performed using EP Evaluator^®^ version 11, Data Innovations LLC (South Burlington, Vermont). Results below the instrument functional sensitivity were handled by using the functional sensitivity divided by the square root of 2. This method has been demonstrated to provide accurate inference [20].

### Results

#### Prolonged storage at − 80 °C does not affect Tg stability

We assessed the impact of storage at − 80 °C for an average of 10.9 months (range: 3.4–15.5 months) on Tg measurement stability [11]. Tg measurements were performed by the EIA DxI 800 analyzers in-house (fresh specimen) and at a reference laboratory (frozen specimen). Within the analytical range of the instruments there was high correlation and no significant sample degradation (Additional file 3: Fig. S1).

#### EIA and LC MS/MS correlation was dependent upon TgAb status and the Tg concentration range

**Fig. 1:**
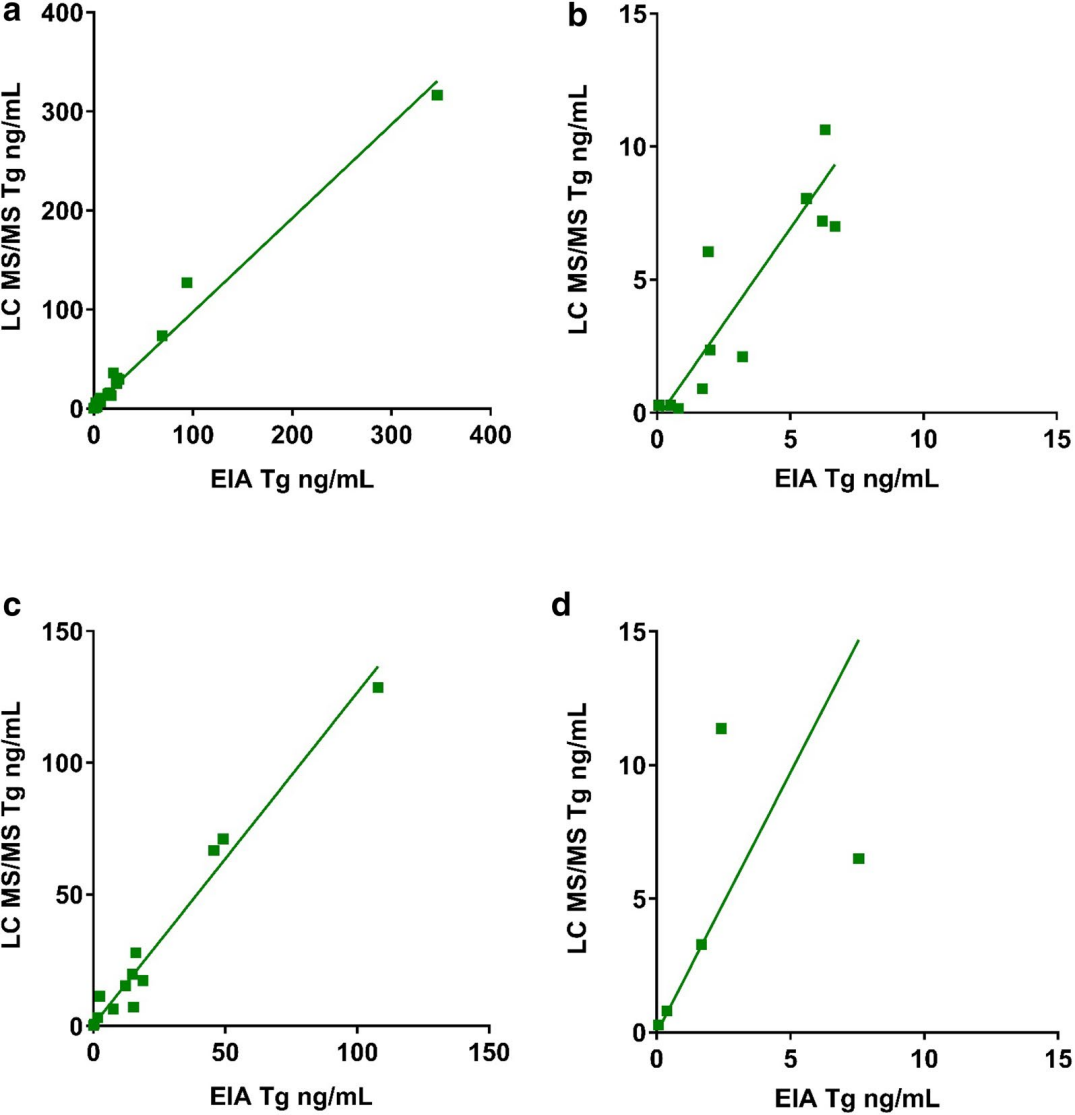
Comparison of Tg measurements by EIA and LC MS/MS methods. **a** TgAb negative samples (N = 28). Tg concentrations (0.07–346.4 ng/mL), (LC MS/MS = 0.95 * EIA + 2.9, r = 0.99, S_yx_ = 8.5). **b** TgAb negative samples (N = 17). Tg concentrations (0.07–6.68 ng/mL), (LC MS/MS = 1.43 * EIA − 0.23, r = 0.92, S_yx_ = 1.5). **c** TgAb positive specimens (N = 20). Tg concentrations (0.07–107.9 ng/mL), LC MS/MS = 1.26 * EIA + 0.45, r = 0.99, S_yx=_ 5.5. **d** TgAb positive specimens (N = 12). Tg concentrations (0.07–7.55 ng/mL), (LC MS/MS = 1.9 * EIA − 0.03, r = 0.68, S_yx_ = 3.3)

Method comparison between the in-house Tg EIA and LC MS/MS methods in TgAb negative specimens correlated well (Fig. 1a). Two specimens were excluded because the reported results were outside the analytical range of the EIA analyzer (837 and 1938 ng/mL). We noted one Tg concentration was significantly higher than the remaining 27 TgAb negative specimens, excluding the potential outlier yielded similar correlation with notable slope bias (LC MS/MS = 1.27 * EIA − 0.80, r = 0.99, S_yx_ = 4.71, N = 27, Additional file 4: Fig. S2). Slope bias was increased in the clinically relevant low Tg concentrations in TgAb negative specimens (Fig. 1b).

Correlation between EIA and LC MS/MS in TgAb positive specimens demonstrated increased slope bias compared to TgAb negative specimens (Fig. 1c). The clinically important lower Tg concentrations in TgAb positive specimens had the lowest correlation coefficient and highest slope bias (Fig. 1d).

Tg concentrations in the healthy volunteer specimens (N = 10) were similar by both the EIA (mean: 19.2, median: 10.6, range: 1.92–94.22 ng/mL) and LC MS/MS (mean: 25.6, median: 11.9, range: 2.4–127.2 ng/mL, LC MS/MS = 1.37 * EIA − 0.57, r = 0.98, S_yx=_ 5.55, Additional file 5 Fig. S3).

#### Reduced correlation of Tg measurements in TgAb positive specimens compared to TgAb negative specimens by EIA and RIA

**Fig. 2:**
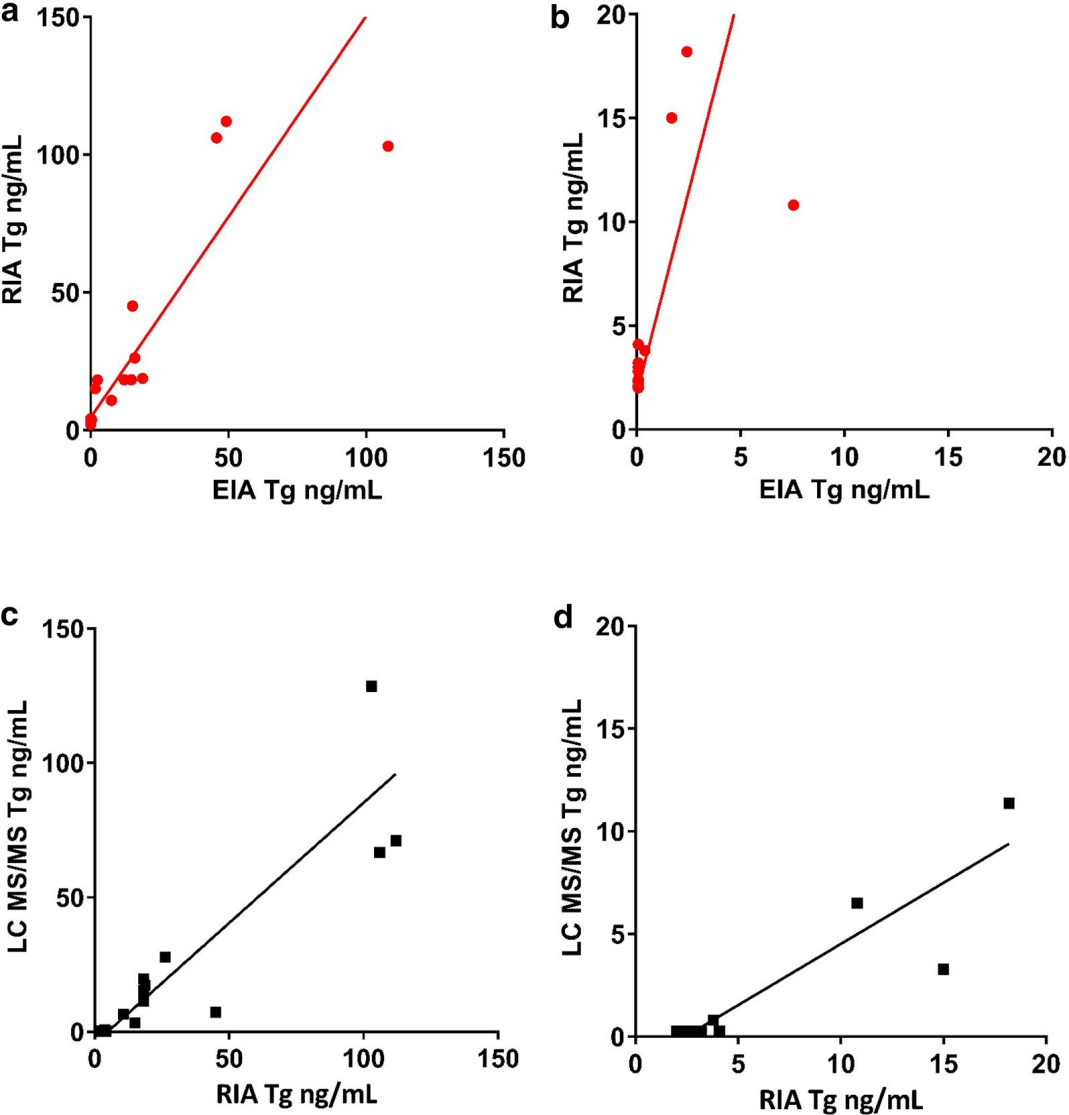
Comparison of Tg measurements in TgAb positive specimens by EIA, RIA, and LC MS/MS methods. **a** TgAb positive specimens (N = 20), EIA Tg concentrations (0.07–107.9 ng/mL), RIA = 1.5 * EIA + 4.6, r = 0.87, S_yx_ = 19.4. **b** TgAb positive samples (N = 12), EIA Tg concentrations (0.07–7.55 ng/mL), RIA = 3.9 * EIA + 1.72, r = 0.60, S_yx_ = 7.2. **c** TgAb positive specimens, N = 20. For EIA Tg concentrations (2.0–112.0 ng/mL), LC MS/MS = 0.89 * RIA − 4.2, r = 0.89, S_yx_ = 14.7. **d** TgAb positive specimens, N = 12. EIA Tg concentrations (2.0–18.2 ng/mL), LC MS/MS = 0.6 * RIA − 1.4, r = 0.90, S_yx_ = 1.6

TgAb can interfere with measurement of Tg by EIA and we refer TgAb positive specimens to a specialized laboratory for Tg analysis by RIA. As expected, we observed reduced correlation between Tg measurements in TgAb positive samples by the Tg EIA and RIA methods (Fig. 2a). Moreover, the correlation was even more reduced at low Tg concentrations (Fig. 2b). Forty percent of the TgAb positive specimens (8 of 20 specimens) had Tg results that were detectable by RIA but undetectable by EIA (Fig. 2).

#### Correlation of Tg measurements in TgAb positive specimens by RIA and LC MS/MS

Comparison of Tg measurements in TgAb positive samples by RIA and LC MS/MS exhibited similar correlation to EIA and RIA (Fig. 2c). At low Tg concentrations the slope of correlation deviated significantly from 1.0 but the correlation coefficient did not (Fig. 2d). Forty percent of the TgAb positive specimens (8 of 20 specimens) had Tg results that were detectable by RIA but undetectable by LC MS/MS (Fig. 2c, d).

#### Analysis of discordant results by chart review

Ten specimens were reported as undetectable or very low by EIA and LC MS/MS but detectable by RIA and we further investigated if the Tg status for these patients agreed with the clinical findings. Tg measurements by the three different methods and clinical follow-up information for these ten patients (average follow-up was 17.4 months) are listed in Table 1. Tg measurements in these patients were performed on samples obtained either at the same time imaging was performed or after. Chart review of these patients suggested that 7 of the 10 specimens would be expected to have had no detectable Tg.

**Table 1:** Clinical Follow-up of Tg concentrations detected by RIA but undetected/low by EIA or LC MS/MS

Sample#	EIA (ng/mL)	RIA (ng/mL)	LC MS/MS (ng/mL)	Thyroid cancer	Subsequent progression	Subsequent Tg measurements	Months monitored	Imaging
26	0	2.8	< 0.4	PTC	No detectable disease	No change	24	Negative
27	0	4.1	< 0.4	PTC (metastatic)	No progression since 2007	No change	24	Negative—neck only
28	0	2.1	< 0.4	PTC	No detectable disease	No change	18	Negative
29	0	2.4	< 0.4	PTC	No detectable disease	No change	14	Negative
30	0	2.3	< 0.4	PTC (metastatic)	No detectable disease	No change	13	Negative
31	0	3	< 0.4	PTC	No detectable disease	No change	17	Negative
32	0	3.2	< 0.4	PTC	Recurrence	No change	11	No progression
33	0	2	< 0.4	PTC	No detectable disease	No change	18	Negative
34	0.39	3.8	0.8	PTC	No detectable disease	No change	18	Remnant
50	1.68	15	3.28	Graves disease	No follow-up for disease	NA	NA	NA

### Discussion

Our current Tg testing algorithm requires determination of TgAb status of specimens with positive TgAb reflexing to an alternate method. Sensitivity is clinically necessary for early recurrence detection in patients followed for DTC. We performed a multi-instrument method comparison study of EIA, RIA and LC MS/MS. Using patient and healthy volunteer samples, we examined Tg measurements in both TgAb negative and positive specimens among the three methods. We found correlation between the in-house EIA and LC MS/MS for Tg measurements in the healthy volunteer specimens. This finding suggests that these methods are likely sensitive enough to distinguish between euthyroid and post-thyroidectomy patients.

TgAb positive specimens with low concentrations of Tg (< 7.55 ng/mL) exhibited reduced correlation among the EIA, RIA and LC MS/MS compared to correlation of all TgAb positive Tg measurements together (Fig. 1c, d). A similar trend was noted for TgAb negative specimen correlation between EIA and LC MS/MS (Fig. 1a, b). This suggests that samples categorized as TgAb negative using a TgAb < 20 IU/mL cutoff may still have appreciable TgAb interference in EIA which is exacerbated at low Tg concentrations. This highlights well-documented issues, by Spencer et al. and others such as the lack of standardization among TgAb methods and the appropriate cutoffs established to ensure TgAb interference is eliminated [8, 10, 11, 13, 21, 22]. The TgAb method used in the study was standardized to the WHO first international reference preparation 65/93 material but TgAb heterogeneity among patients may account for the variability in measurement [8, 10].

In TgAb positive specimens tested by EIA and RIA, due to TgAb interference, we saw discrepancy in Tg measurements (Fig. 2). Correlation between RIA and LC MS/MS Tg measurements demonstrated some discrepancies which appeared to be due to Tg measured by LC MS/MS that was undetectable or low for 10 of 20 specimens but detectable at appreciable concentrations by RIA (Fig. 2; Table 1). Seven of the individuals with discrepant LC MS/MS and RIA Tg concentrations had no evidence of disease for approximately 1 year after this specimen was analyzed. This finding is not without precedence [16, 19, 23–25]. It is unclear if this discrepancy is due to Tg overestimation by RIA due to TgAb interference which has been well demonstrated previously [16, 19, 24, 25] or failure of the LC MS/MS method to detect the Tg due to a polymorphism [23]. These data demonstrate that it is vital that Tg measurements be performed by the same method during clinical follow-up. Separate analysis of low and high Tg concentrations may be appropriate for method validations. Understanding of the correlations at low concentrations was important for our clinicians’ evaluation of this testing as the majority of Tg testing in our patient population is follow-up of post thyroidectomy DTC patients.

Clinicians involved in the care of DTC patients have noted that once TgAb are detected in the specimen they interpret the Tg result as unreliable. Despite this distrust of the Tg measurements, this test was ordered on each of these patients at every visit. Within our system clinicians are not yet prepared to change from RIA to LC–MS/MS despite the known TgAb interference issues with RIA. While 7 of the 10 discrepant cases indicated no disease progression there was concern that early recurrence may be missed by LC–MS/MS and follow-up of falsely elevated Tg is preferable to delays in recurrence detection.

## Limitations

For the Tg method comparison study between the RIA and LC MS/MS method, we only examined TgAb positive specimens for logistical reasons. Since we did not compare Tg measurements in TgAb negative specimens between RIA and LC MS/MS, we are unable to determine if the positive bias exhibited by the RIA compared to the LC MS/MS is due to TgAb interference, calibration, or reagent differences. Our 11–24 month follow-up of 10 discrepant Tg concentrations suggested that 7 of the 10 RIA Tg concentrations are not associated with disease recurrence. However, follow-up that ranges over a longer time period, 5 years or more, will be more definitive.

Our study is indicative of the studies performed by individual clinical laboratories to verify and investigate potential new methodologies and is therefore a small cohort study.

## Additional files

**Additional file 1: Table S1.** Subject socio-demographics and clinical characteristics.

**Additional file 2: Table S2.** Thyroglobulin and thyroglobulin antibody methodologies.

**Additional file 3: Figure S1.** Short term freezer storage does not affect Tg stability.

**Additional file 4: Figure S2.** Comparison of Tg measurements by EIA and LC MS/MS methods for low TgAb excluding potential outlier at 346.4 ng/mL.

**Additional file 5: Figure S3.** Tg measurements in healthy volunteers.

## Abbreviations

Tg: thyroglobulin
TgAb: thyroglobulin antibody
EIA: enzyme immunoassay
RIA: radioimmunoassay
LC–MS/MS: liquid chromatography tandem mass spectrometry
DTC: differentiated thyroid cancer
PTC: papillary thyroid cancer
FTC: follicular thyroid cancer
