# Supplementary material for: Clinical laboratory verification of thyroglobulin concentrations in the presence of autoantibodies to thyroglobulin: comparison of EIA, radioimmunoassay and LC MS/MS measurements in an Urban Hospital
Source: BMC Res Notes. 2017 Dec 8;10:725. doi: 10.1186/s13104-017-3050-6 (PMC5723050; doi:10.1186/s13104-017-3050-6)
Supplement: Supplementary file 1 — Additional file 1: Table S1. Subject socio-demographics and clinical characteristics. [file 13104_2017_3050_MOESM1_ESM.pptx]

## Slide 1
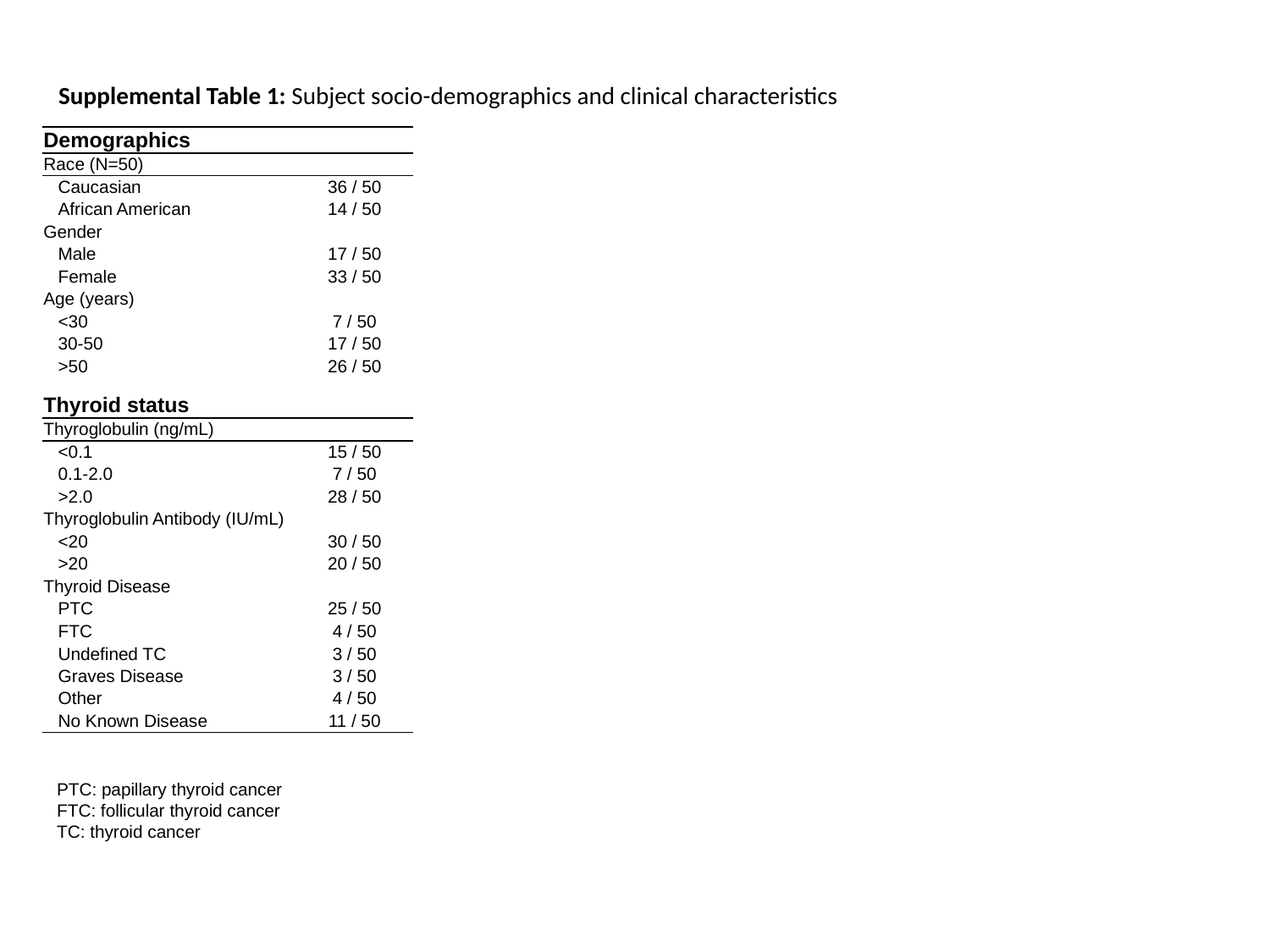

Supplemental Table 1: Subject socio-demographics and clinical characteristics
| Demographics | |
| --- | --- |
| Race (N=50) | |
| Caucasian | 36 / 50 |
| African American | 14 / 50 |
| Gender | |
| Male | 17 / 50 |
| Female | 33 / 50 |
| Age (years) | |
| <30 | 7 / 50 |
| 30-50 | 17 / 50 |
| >50 | 26 / 50 |
| | |
| Thyroid status | |
| Thyroglobulin (ng/mL) | |
| <0.1 | 15 / 50 |
| 0.1-2.0 | 7 / 50 |
| >2.0 | 28 / 50 |
| Thyroglobulin Antibody (IU/mL) | |
| <20 | 30 / 50 |
| >20 | 20 / 50 |
| Thyroid Disease | |
| PTC | 25 / 50 |
| FTC | 4 / 50 |
| Undefined TC | 3 / 50 |
| Graves Disease | 3 / 50 |
| Other | 4 / 50 |
| No Known Disease | 11 / 50 |
PTC: papillary thyroid cancer
FTC: follicular thyroid cancer
TC: thyroid cancer
