# Supplementary material for: Clinical laboratory verification of thyroglobulin concentrations in the presence of autoantibodies to thyroglobulin: comparison of EIA, radioimmunoassay and LC MS/MS measurements in an Urban Hospital
Source: BMC Res Notes. 2017 Dec 8;10:725. doi: 10.1186/s13104-017-3050-6 (PMC5723050; doi:10.1186/s13104-017-3050-6)
Supplement: Supplementary file 2 — Additional file 2: Table S2. Thyroglobulin and thyroglobulin antibody methodologies. [file 13104_2017_3050_MOESM2_ESM.pptx]

## Slide 1
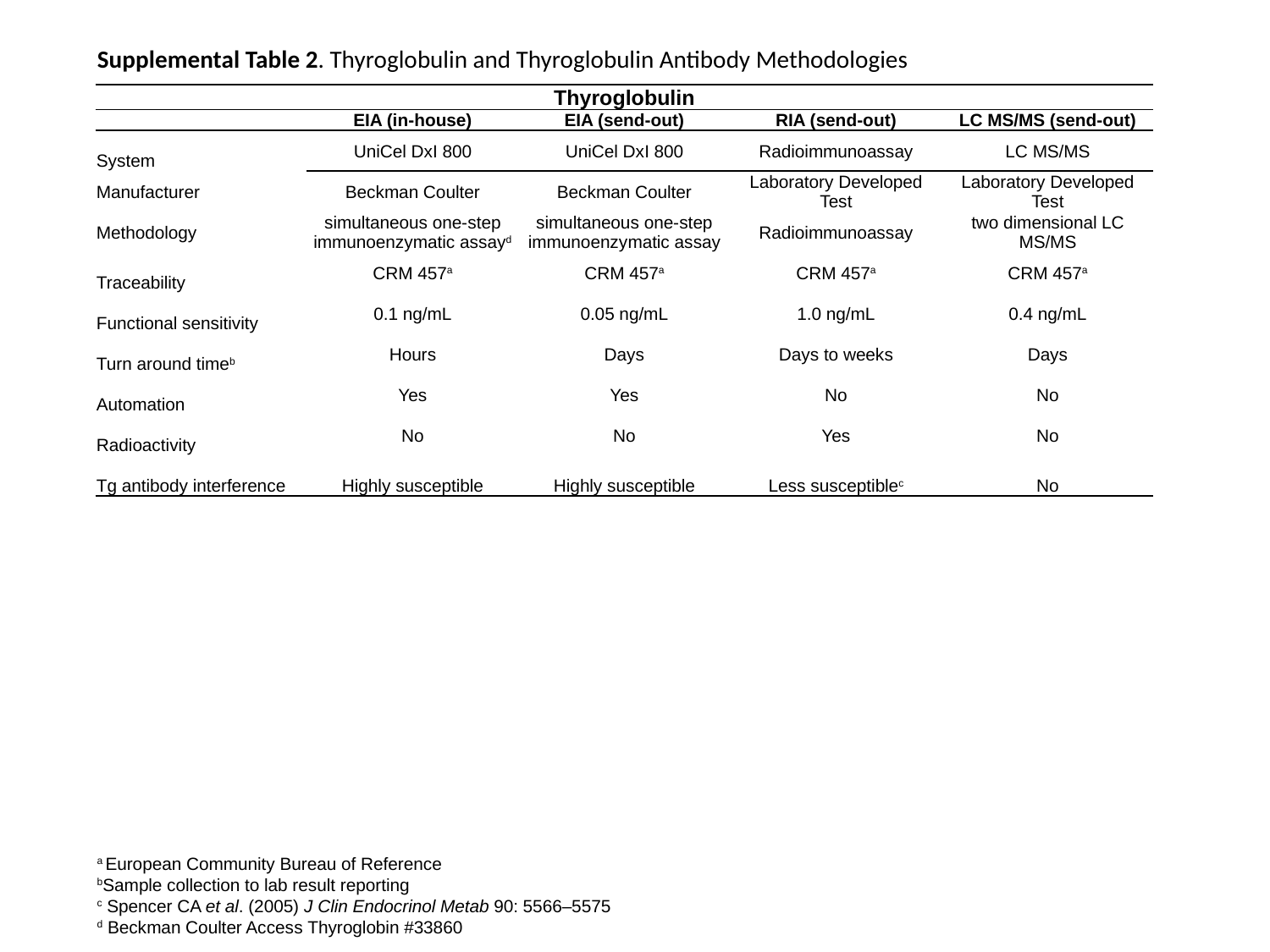

# Supplemental Table 2. Thyroglobulin and Thyroglobulin Antibody Methodologies
| Thyroglobulin | | | | |
| --- | --- | --- | --- | --- |
| | EIA (in-house) | EIA (send-out) | RIA (send-out) | LC MS/MS (send-out) |
| System | UniCel DxI 800 | UniCel DxI 800 | Radioimmunoassay | LC MS/MS |
| Manufacturer | Beckman Coulter | Beckman Coulter | Laboratory Developed Test | Laboratory Developed Test |
| Methodology | simultaneous one-step immunoenzymatic assayd | simultaneous one-step immunoenzymatic assay | Radioimmunoassay | two dimensional LC MS/MS |
| Traceability | CRM 457a | CRM 457a | CRM 457a | CRM 457a |
| Functional sensitivity | 0.1 ng/mL | 0.05 ng/mL | 1.0 ng/mL | 0.4 ng/mL |
| Turn around timeb | Hours | Days | Days to weeks | Days |
| Automation | Yes | Yes | No | No |
| Radioactivity | No | No | Yes | No |
| Tg antibody interference | Highly susceptible | Highly susceptible | Less susceptiblec | No |
a European Community Bureau of Reference
bSample collection to lab result reporting
c Spencer CA et al. (2005) J Clin Endocrinol Metab 90: 5566–5575
d Beckman Coulter Access Thyroglobin #33860
