# Supplementary material for: Clinical laboratory verification of thyroglobulin concentrations in the presence of autoantibodies to thyroglobulin: comparison of EIA, radioimmunoassay and LC MS/MS measurements in an Urban Hospital
Source: BMC Res Notes. 2017 Dec 8;10:725. doi: 10.1186/s13104-017-3050-6 (PMC5723050; doi:10.1186/s13104-017-3050-6)
Supplement: Supplementary file 3 — Additional file 3: Figure S1. Short term freezer storage does not affect Tg stability. [file 13104_2017_3050_MOESM3_ESM.pptx]

## Slide 1
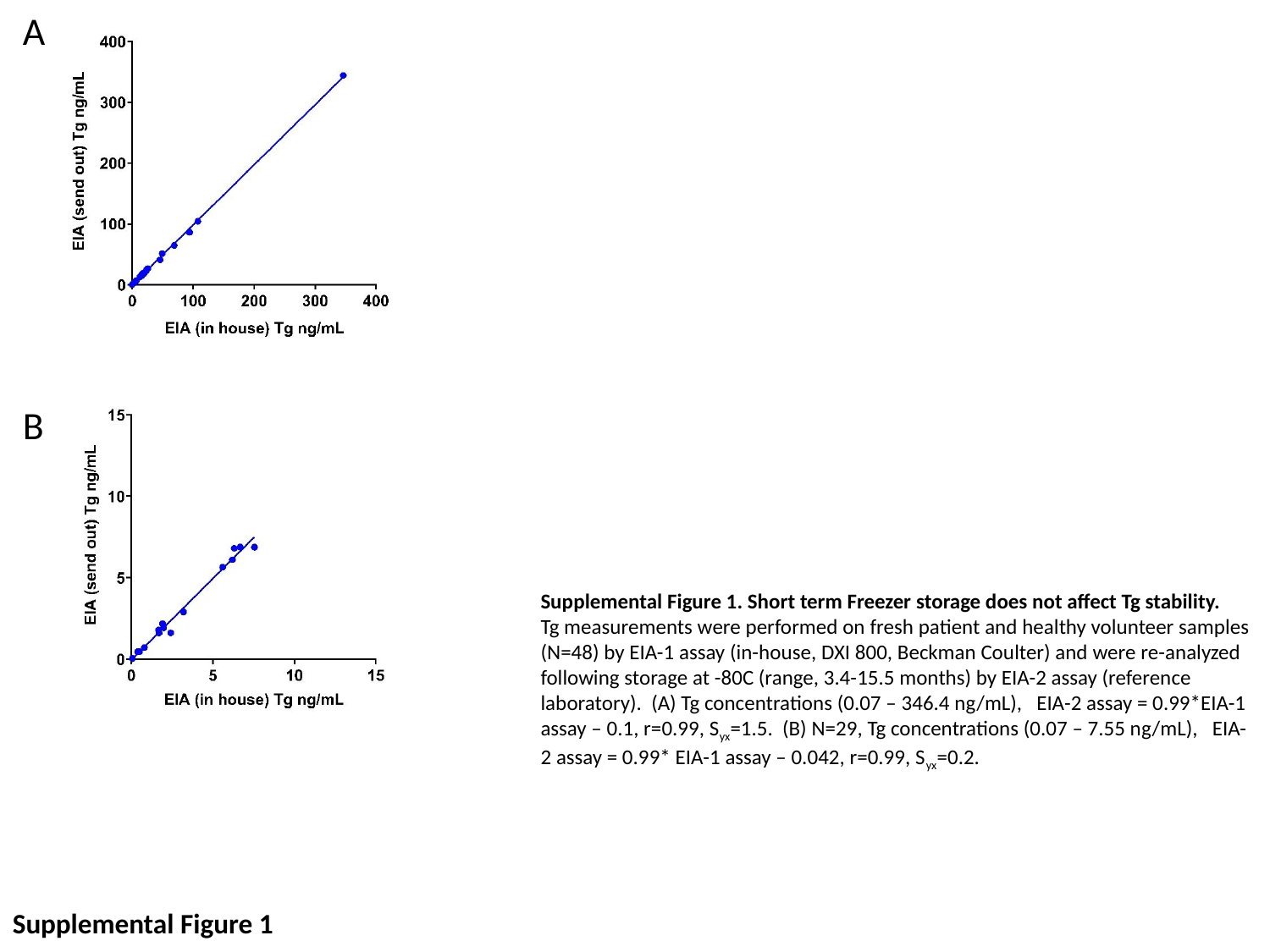

A
B
Supplemental Figure 1. Short term Freezer storage does not affect Tg stability.
Tg measurements were performed on fresh patient and healthy volunteer samples (N=48) by EIA-1 assay (in-house, DXI 800, Beckman Coulter) and were re-analyzed following storage at -80C (range, 3.4-15.5 months) by EIA-2 assay (reference laboratory). (A) Tg concentrations (0.07 – 346.4 ng/mL), EIA-2 assay = 0.99*EIA-1 assay – 0.1, r=0.99, Syx=1.5. (B) N=29, Tg concentrations (0.07 – 7.55 ng/mL), EIA-2 assay = 0.99* EIA-1 assay – 0.042, r=0.99, Syx=0.2.
Supplemental Figure 1
