# Supplementary material for: Clinical laboratory verification of thyroglobulin concentrations in the presence of autoantibodies to thyroglobulin: comparison of EIA, radioimmunoassay and LC MS/MS measurements in an Urban Hospital
Source: BMC Res Notes. 2017 Dec 8;10:725. doi: 10.1186/s13104-017-3050-6 (PMC5723050; doi:10.1186/s13104-017-3050-6)
Supplement: Supplementary file 4 — Additional file 4: Figure S2. Comparison of Tg measurements by EIA and LC MS/MS methods for low TgAb excluding potential outlier at 346.4 ng/mL. [file 13104_2017_3050_MOESM4_ESM.pptx]

## Slide 1
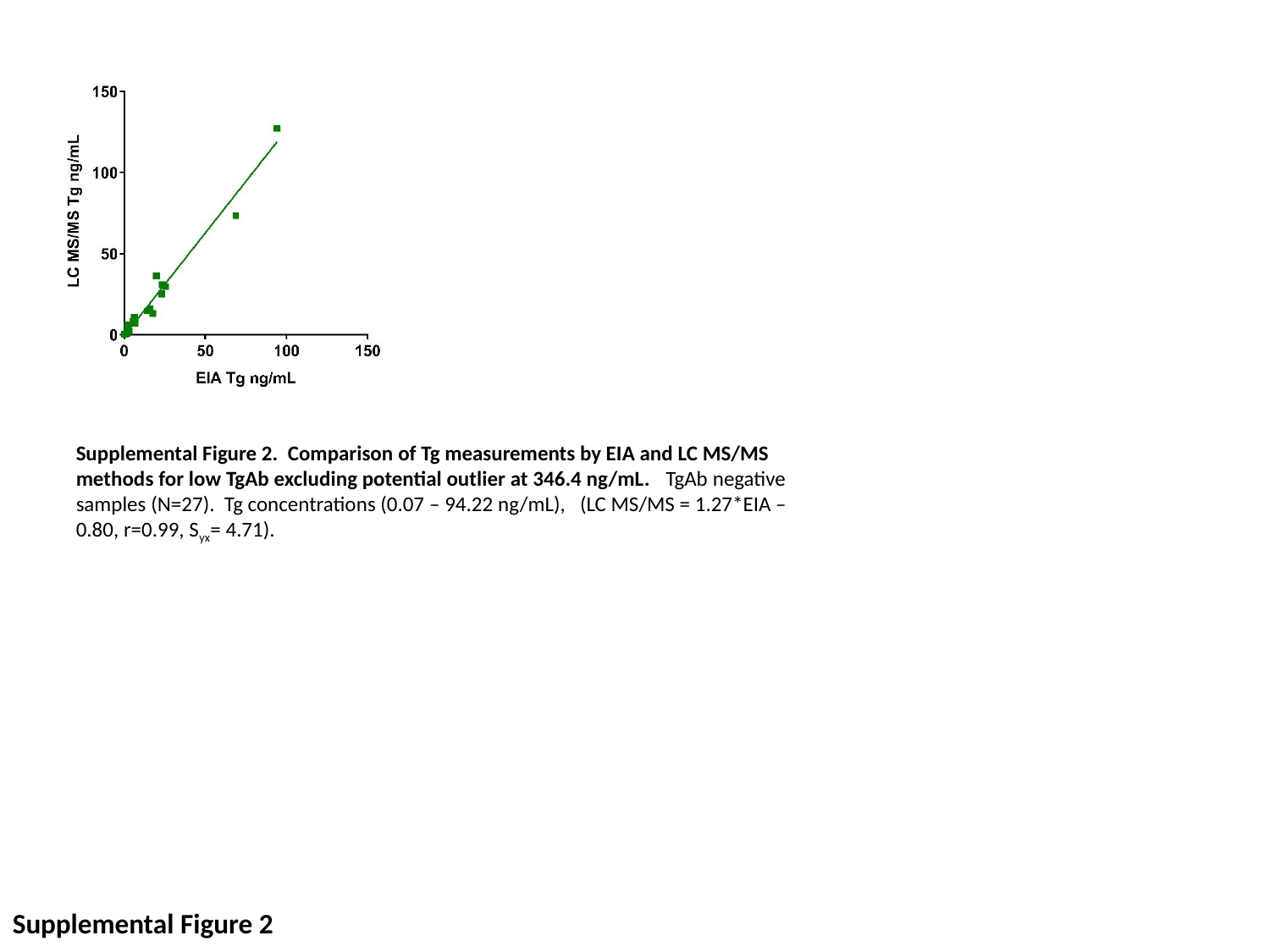

Supplemental Figure 2. Comparison of Tg measurements by EIA and LC MS/MS methods for low TgAb excluding potential outlier at 346.4 ng/mL. TgAb negative samples (N=27). Tg concentrations (0.07 – 94.22 ng/mL), (LC MS/MS = 1.27*EIA – 0.80, r=0.99, Syx= 4.71).
Supplemental Figure 2
