# Supplementary material for: Clinical laboratory verification of thyroglobulin concentrations in the presence of autoantibodies to thyroglobulin: comparison of EIA, radioimmunoassay and LC MS/MS measurements in an Urban Hospital
Source: BMC Res Notes. 2017 Dec 8;10:725. doi: 10.1186/s13104-017-3050-6 (PMC5723050; doi:10.1186/s13104-017-3050-6)
Supplement: Supplementary file 5 — Additional file 5: Figure S3. Tg measurements in healthy volunteers. [file 13104_2017_3050_MOESM5_ESM.pptx]

## Slide 1
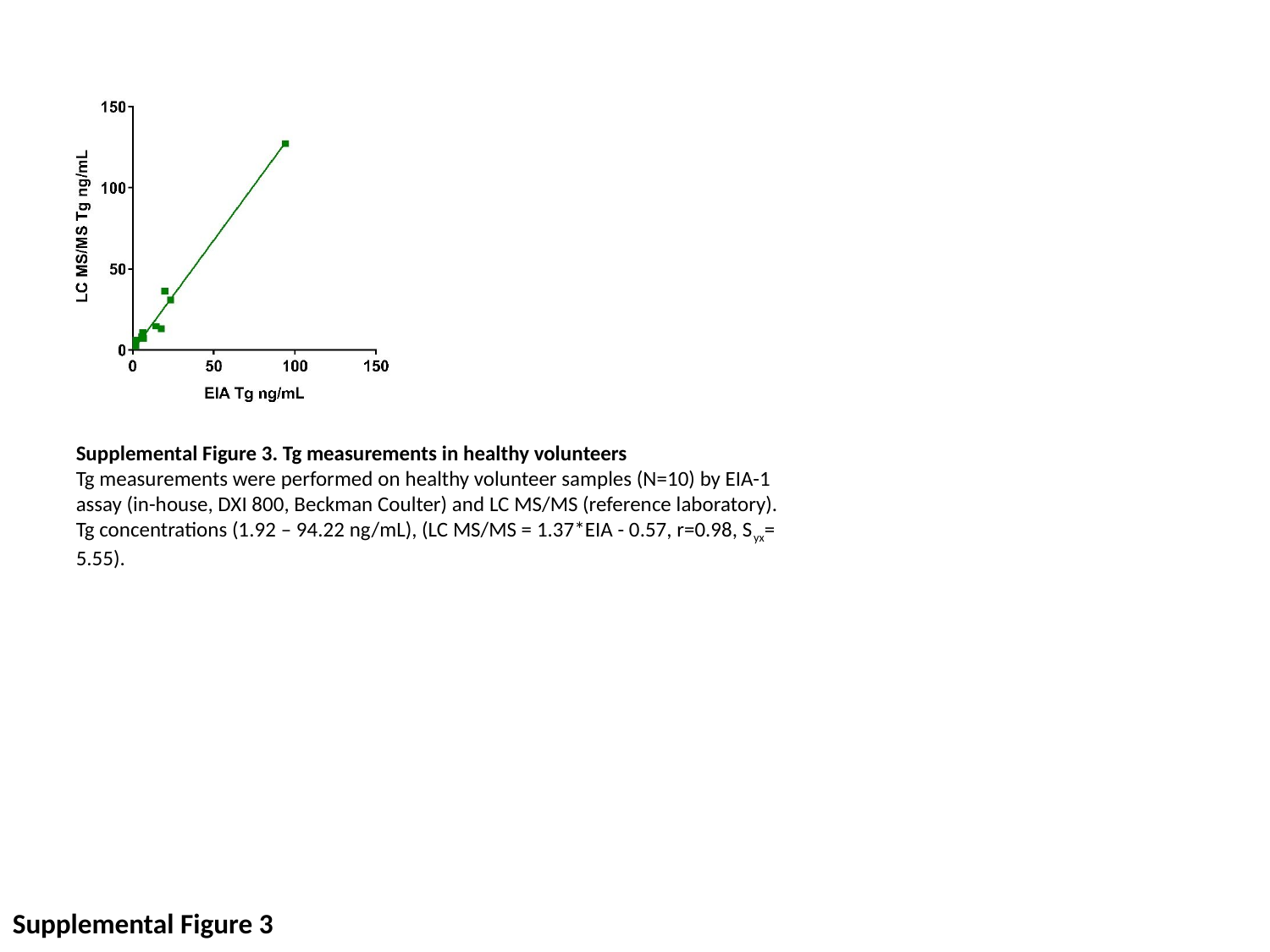

Supplemental Figure 3. Tg measurements in healthy volunteers
Tg measurements were performed on healthy volunteer samples (N=10) by EIA-1 assay (in-house, DXI 800, Beckman Coulter) and LC MS/MS (reference laboratory). Tg concentrations (1.92 – 94.22 ng/mL), (LC MS/MS = 1.37*EIA - 0.57, r=0.98, Syx= 5.55).
Supplemental Figure 3
